# Is Sedentary Behavior Associated With Executive Function in Children and Adolescents? A Systematic Review

**DOI:** 10.3389/fpubh.2022.832845

**Published:** 2022-02-02

**Authors:** Shiyuan Li, Jinyang Guo, Kefeng Zheng, Mengyao Shi, Tao Huang

**Affiliations:** ^1^Department of Physical Education, Shanghai Jiao Tong University, Shanghai, China; ^2^School of Education, Shanghai Jiao Tong University, Shanghai, China

**Keywords:** sedentary behavior, screen time, executive function, children, adolescents

## Abstract

**Background:**

Prolonged time on sedentary behavior, especially screen-based sitting time, is associated with unfavorable health indicators in children and adolescents. However, the effects of sedentary behavior on cognitive function remain to be elucidated.

**Objective:**

The purpose of this systematic review was to synthesize the evidence on the associations of sedentary behavior with executive function in children and adolescents.

**Methods:**

Four electronic databases (i.e., PubMed, Web of Science, PsycINFO, and SPORTDiscus) were searched for studies examining the associations between sedentary behavior and executive function in children and adolescents. Study quality was assessed by the NIH Quality Assessment Tool for Observational Cohort and Cross-Sectional Studies.

**Results:**

A total of 1,151 records were initially identified through database searches and other searches. Twelve cross-sectional and four longitudinal studies met the inclusion criteria. Of the 16 studies, seven studies found significant negative associations between sedentary behavior and executive function, and two studies presented positive associations. Eight studies measured sedentary time using accelerometers and showed varied associations between objectively measured sedentary time and executive function. Nine studies measured screen-based sedentary behavior, of which five studies found negative associations of sedentary time with executive function.

**Conclusion:**

The available evidence on the associations between sedentary behavior and executive function is not conclusive in children and adolescents. However, screen-based sedentary behavior may be negatively associated with executive function.

## Introduction

Sedentary behavior is a distinct construct from physical activity, referring to any waking behaviors with an energy expenditure of <1.5 metabolic equivalent units (METs) while in a sitting, reclining, or lying posture (1). Common sedentary behaviors include prolonged sitting, screen-based behaviors (e.g., TV viewing, computer/tablet using, video gaming), etc. Time spent on sedentary behaviors can be self/parent-reported or be objectively monitored by wearable devices such as accelerometers. Currently, sedentary time remains high in children and adolescents, and the trend continues to increase over the past few decades in some countries. According to the Global School-based Student Health Survey among 97 countries, about 25% of boys and girls aged 13–15 years old reported sitting longer than 3 h per day, in addition to sitting at school and for homework (2). From 2007 to 2016, the estimated total sitting time increased from 7 h per day to 8.2 h per day among adolescents in the United States (3). In China, an increasing trend of the prevalence of screen-based viewing time was also observed in school-age children (4).

Accumulating evidence showed that sedentary behavior, especially prolonged TV viewing, have been linked with increased risk of a variety of chronic diseases, such as obesity (5), type 2 diabetes (6, 7), cardiovascular diseases (8), and certain types of cancer (9). In children and adolescents, sedentary behaviors have also been linked with unfavorable health indicators, such as lower physical fitness (10), higher fatness (11), clustered cardiometabolic risk scores (12), and lower self-esteem (13). Even worse, emerging evidence has shown that excessive sedentary behaviors are associated with mental illness and poorer cognitive function (14, 15). However, the findings on the relationship between sedentary behavior and cognitive function are mixed. A systematic review included eight studies examining the associations of sedentary behavior with cognitive function in adults older than 40 years (15). It concluded that greater amounts of sedentary behaviors were associated with poorer cognitive function over the lifespan. A more recent systematic review including 13 cross-sectional and longitudinal studies suggested inconsistent evidence on the direction of the association of sedentary behavior with cognitive function in older adults with a mean age of 65+ years (16). Another systematic review in young children (≤5 years) found that different types of sedentary behavior may exert different influences on cognitive development (17). Screen time, particularly TV viewing, was either not associated with or negatively associated with cognitive skills. However, no existing studies have critically reviewed the literature of the association between sedentary behavior and executive function in children and adolescents.

Previous studies have shown that physical activity and fitness have beneficial effects on cognitive function in children and adolescents (18, 19). The effects are disproportionately larger for executive function (20). Executive function refers to a set of top-down mental processes needed for goal-directed behaviors, such as inhibitory control, working memory, cognitive flexibility, planning (21). Executive function is critical for school readiness, academic performance, and future career success (21, 22). However, evidence on the effects of sedentary behavior on executive function remains conflicting in children and adolescents. Some studies showed that sedentary behavior is not associated with executive function in childhood (23–25), whereas other studies found negative (26, 27) or positive associations (28). No existing systematic reviews have addressed this research gap. In addition, a preview review suggested a type-specific association between sedentary behavior and health indicators in children and adolescents (29). Another gap in the literature is the lack of the associations between type-specific sedentary behaviors and executive function.

Therefore, the current systematic review is aimed to synthesize the literature on the association of sedentary behavior (both objectively measured sedentary time and self/parent-reported screen-based behaviors) with executive function in children and adolescents.

## Methods

This systematic review was performed following the Preferred Reporting Items for Systematic Reviews and Meta-Analyses (PRISMA) statement (30).

### Search Strategy

Two authors (SL and JG) independently searched PubMed, Web of Science, PsycINFO, and SPORTDiscus from inception to April 2021. The combinations of the following three groups of retrieval items were used: (1) sedentary behavi^*^, screen time, sitting time, sedentary time, TV viewing, video gam^*^, computer use; (2) executive function, cognitive control, working memory, inhibitory control, cognitive flexibility, planning; (3) children, adolescents. The specific search strategy was slightly adjusted according to the search builder of each database.

### Study Selection

Two authors (SL and JG) screened all the retrieved titles and abstracts to exclude duplicate or irrelevant studies. The two authors screened the full text of the remaining studies after removing duplicate and apparently irrelevant studies. Any disagreements about the study selection were discussed with a third author (TH) until a consensus was reached. The two review authors also searched the bibliographies of all included articles to ensure that all relevant studies were captured. Only the longitudinal result was extracted for the cohort studies that conducted both cross-sectional and longitudinal analyses in the same population.

### Inclusion and Exclusion Criteria

The studies must meet the following inclusion criteria to be included: (1) studies with cross-sectional or longitudinal design examined the associations between sedentary behavior and executive function; (2) sedentary behavior was self/parent-reported (e.g., prolonged sitting, TV viewing, computer use, video gaming) or objectively monitored by wearable devices (e.g., accelerometers); (3) executive function was objectively assessed (paradigms including Flanker task, Stroop color-word test, N-back task, Tower of London task, Trail making task, etc.); (4) the participants were apparently healthy children and adolescents aged 5–17 years; (5) studies must be published in peer-reviewed journals; (6) English full text must be available.

Studies were excluded if the sedentary behaviors were not clearly classified or measured. Studies focusing on specific screen-based or non-screen contents (e.g., violent films, educational programs), screen-based active behavior (e.g., active video gaming), or specific learning behavior (e.g., reading, puzzles) were excluded. Studies were also excluded if the executive function was parent- or teacher-reported.

### Data Extraction

Data collection was conducted independently by two authors (SL and JG). Publication year, country, study design, sample size, covariates, measurement of sedentary behavior, assessment of executive function, and results were extracted from each included study and recorded.

### Methodological Quality

The two authors assessed the quality of studies by the NIH Quality Assessment Tool for Observational Cohort and Cross-Sectional Studies (31). This tool includes 14 items, and the reviewer could select “yes,” “no,” “cannot determine,” or “not reported” on each item. The score for longitudinal studies ranges from 0 (the lowest quality) to 14 (the highest quality). For cross-sectional studies, three items are not applicable (items 10, 12, 13). The classifications of methodological quality are rated as “strong” (≥80%), “good” (70–79%), “fair” (60–69%), or “poor” (<60%) based on the percentage scores which are calculated as the number of “yes” responses divided by the total number of applicable items (32, 33). All discrepancies between reviewers were resolved through discussion among the reviewers or with a third reviewer if needed. The items of assessment tool are listed in Supplementary Table 1.

## Results

### Search Results and Study Characteristics

**Figure 1:**
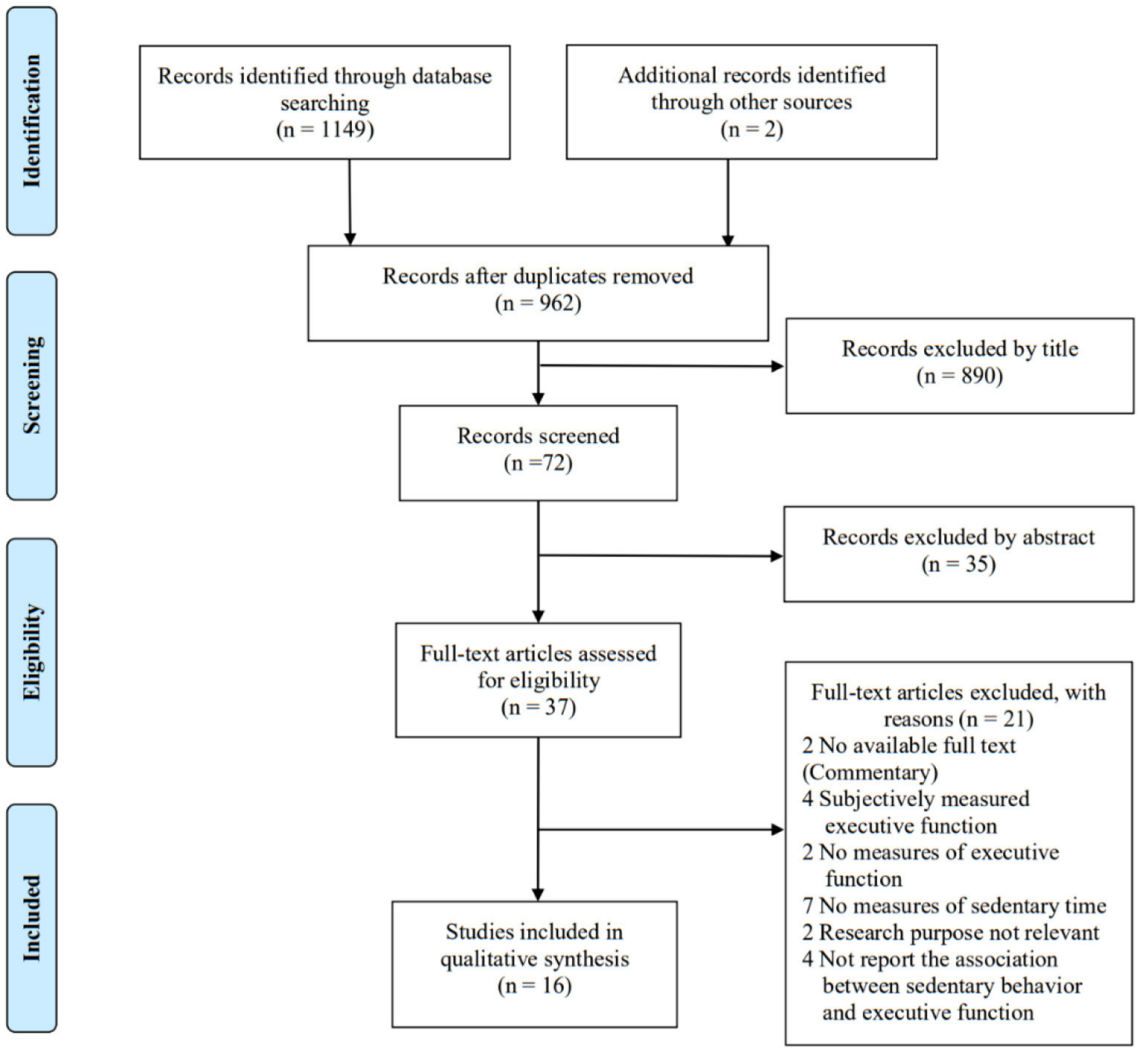
PRISMA flow diagram of study selection.

A total of 1,149 records were identified through database searches, and additional two records were identified through reference list searches (see Figure 1). After removing duplicate records, 962 records remained. Following the screening of titles and abstracts, 37 articles were obtained for further full text review. Sixteen articles met the inclusion criteria after detailed assessment of the full-text, including 12 cross-sectional studies and four longitudinal studies.

Sedentary behaviors were measured by subjective assessment (self-reported or parent-reported) and objective assessment (accelerometer). Eight studies objectively measured sedentary time using accelerometers (25, 27, 28, 34–38). Nine studies surveyed a variety of screen-based behaviors as proxies of sedentary behaviors (i.e., total screen time, TV viewing, computer gaming, other computer use, general computer use, etc.) (23, 24, 26, 37, 39–43).

Across these 16 studies, a total of 21 cognitive tasks were used, measuring various aspects of executive function, including inhibitory control, working memory, cognitive flexibility, and planning. Study sample sizes ranged from 77 to 1,001. The participants were aged 5–17 years old. The countries of study locations were Norway, China, South Africa, Canada, Spain, United States, United Kingdom, Finland, and the Netherlands.

### Study Findings

Of the included 16 studies with objectively measured sedentary time or screen-based sedentary behavior, seven studies (44%) found a negative association between sedentary behavior and executive function (23, 24, 26, 27, 40, 41, 43), while two studies (13%) found a positive association between sedentary bahavior and executive function (28, 34).

#### Objectively Measured Sedentary Time and Executive Function

Of the eight studies with objectively measured sedentary time (25, 27, 28, 34–38), one study (13%) found that more sedentary time was associated with poorer inhibitory control (27). Two of the eight studies (25%) demonstrated a positive association of sedentary time with one or more aspects of executive functions (inhibitory control, working memory, and planning) (28, 34), including one longitudinal study (28). Six studies (75%) observed no associations between objectively measured sedentary time and certain aspects of executive functions (25, 27, 35–38).

#### Screen-Based Sedentary Behavior and Executive Function

Nine studies investigated the associations of screen-based sedentary behaviors (i.e., total screen time, TV viewing, computer/video gaming, other computer use) with executive function (23, 24, 26, 37, 39–43). Of the nine studies, five studies (56%) found negative associations between screen-based sedentary behavior and certain aspects of executive function (26, 37, 40, 41, 43). Of note, one of them employed longitudinal study design (26). Eight studies (89%) observed no associations between screen-based sedentary behavior and certain aspects of executive functions (23, 24, 26, 37, 39–42).

Of the nine studies, three studies assessed the total screen time (26, 37, 39). Two of these studies (66%) showed that total screen time was not associated with executive function (working memory, cognitive flexibility) (37, 39). Only one study (33%) observed a negative association between total screen time and N-back performance in girls (26). Seven studies examined the associations between TV viewing and executive function (23, 24, 26, 37, 40, 41, 43). Of them, two studies (29%) found that more time on TV viewing was associated with poorer executive function (40, 43), and five studies (71%) did not find any associations between TV viewing and executive function (23, 24, 26, 37, 41). Three studies examined the association between general computer usages with executive function (40–42). One study found a positive association (40) and one study found a negative association (41). Two studies examined the associations between computer/video gaming and executive function (26, 37). Both of them found that spending more time on computer/video gaming was related to worse working memory.

### Methodological Quality of Included Studies

The average score of cross-sectional studies was 6.67 (Table 1). The average score of longitudinal studies was 10. Detailed scores of quality assessment are also available in Supplementary Table 1.

**Table 1:** Characteristics and results table for included studies.

**References, Country**	**Sample**	**Measurement of sedentary behavior**	**Measurement of executive function**	**Adjusted covariates**	**Results**
	**(1)** ***N*** **(% girls)** **(2) Age (years)**				
**Cross-sectional studies**					
Aadland et al. (34) Norway	(1) 697 (51%) (2) 10.2 ± 0.3	Accelerometer -Sedentary time	Stroop color-word test -Inhibitory control Digit span test -Working memory Verbal fluency & Trail making test -Cognitive flexibility	Age; body fat; pubertal status; birth weight; SES	Time on sedentary behavior was positively associated with working memory in girls, but with inhibitory control and cognitive flexibility in boys.
Chetty-Mhlanga et al. (39) South Africa	(1) 1,001 (53%) (2) 11 ± 1.7	Self-reported -Total screen time	Spatial working memory test (CANTAB) -Working Memory Multi-tasking test (CANTAB) -Cognitive flexibility	Age; sex; area; head injury; smoke; alcohol; drugs; farm residence; SES; mobile phone ownership; mother employment; mother education; home language; household size; government grant; repeated grade	Total screen time was not associated with working memory and cognitive flexibility.
Fairclough et al. (38) United Kingdom	(1) 359 (51%) (2) 11.5 ± 1.4	Accelerometer -Sedentary time	Spatial working memory test (CANTAB) -Working memory Multi-tasking test (CANTAB) -Inhibitory control Intra-Extra dimensional set shift task (CANTAB) -Cognitive flexibility	Age; sex; BMI z-score; IMD decile	Sedentary time was not associated with inhibitory control and cognitive flexibility.
Mora-Gonzalez et al. (25) Spain	(1) 79 (45%) (2) 10.2 ± 1.1	Accelerometer -Sedentary time	Delayed non-matched-to-sample task -Working memory	Sex; age; wave of participation; peak height velocity; BMI; parent education; IQ	Sedentary time was not associated with working memory.
Mora-Gonzalez et al. (35) Spain	(1) 100 (42%) (2) 10.1 ± 1.1	Accelerometer -Sedentary time	Stroop color-word test -Inhibitory control Zoo map task -Planning Design Fluency test and Trail making task -Cognitive flexibility	Sex; peak height velocity; BMI; wave of participation; parent education; IQ; MVPA	Sedentary time was not associated with inhibitory control, planning, and cognitive flexibility.
Mora-Gonzalez et al. (36) Spain	(1) 84 (44%) (2) 10.1 ± 1.1	Accelerometer -Sedentary time	Flanker task -Inhibitory control	Sex; peak height velocity; BMI; parent education; IQ	Sedentary time was not associated with inhibitory control.
Ribner et al. (43) United States	(1) 807 (50%) (2) 5.7 ± 0.3	Parent-reported -TV viewing	Hearts and flowers task Dimensional change card sort Flanker task -Working memory -Cognitive flexibility -Inhibitory control	Age; sex; performance on Raven's progressive matrices	TV viewing was negatively associated with composite executive function.
Rosenqvist et al. (40) United States	(1) 381 (55%) (2) 8.4 ± 2.3	Parent-reported -TV viewing -General computer use	NEPSY-II -Inhibitory control	Age; sex; maternal education; other media variables	General computer use was not associated with inhibitory control. Negative association was observed between TV viewing and inhibitory control.
Syvaoja et al. (37) Finland	(1) 224 (57%) (2) 12.2 ± 0.6	Self-reported -TV viewing -Computer/video gaming -Computer use (other than playing) Accelerometer -Sedentary time	Spatial span test -Working memory Intra-Extra dimensional set shift task -Cognitive flexibility	Parental education; remedial education; gender; MVPA	Objective sedentary time, total screen time or TV viewing were not associated with any measures of executive functions. Computer/video game playing was negatively associated with working memory, but not with cognitive flexibility. Computer use was negatively associated with cognitive flexibility.
van der Niet et al. (27) Netherland	(1) 77 (55%) (2) 8.9 ± 1.0	Accelerometer -Sedentary time	Stroop color-word test -Inhibitory control Visual memory span test -Working memory Trail making task -Cognitive flexibility Tower of London task -Planning	Sex; age; SES	More time spent in sedentary behavior was associated with worse inhibitory control, but not to other aspects of executive functions.
Verburgh et al. (41) Netherland	(1) 168 (0%) (2) 8–12	Self-reported -TV viewing -General Computer use	Stop signal task -Inhibitory control Digit span task -Working memory Flanker Task -Executive attention	Age; BMI; IQ	General computer use was negatively associated with inhibitory control, but not with working memory and cognitive flexibility. TV viewing was not associated with any aspects of executive functions.
Xu et al. (42) United Kingdom and China	(1) 371 (47%) (2) 12.2 ± 1.0	Self-reported -General computer use	Stop signal task -Inhibitory control Figure matching task -Cognitive flexibility Spatial span task -Working memory Tower of Hanoi task -Planning	Age; general cognitive ability; family SES	General computer use was not associated with any aspects of executive functions.
**Longitudinal studies**					
Dubuc et al. (26) Canada	(1) 187 (62%) (2) baseline age: 13.1 ± 1.0 Follow-up age: 16.1	Self-reported -Total screen time -TV viewing -Computer/video gaming -Computer use (other than game playing)	Flanker task -Inhibitory control N-back task -Working memory	Age; pubertal status; socioeconomic status; ethnicity	In female students, changes in total screen time and time on video games were negatively associated with changes in N-back accuracy. In male students, changes in screen time was not associated with performance on Flanker task and N-back task.
López-Vicente et al. (24) Spain	(1) 307 (51%) (2) baseline age: 6 follow-up age: 14	Parent reported -TV viewing -Other sedentary behaviors	N-back task -Working memory	Age; sex; maternal education	TV viewing was not associated with working memory. Other sedentary behaviors were negatively associated with working memory.
O'Connor et al. (23) Spain	(1) 278 (49%) baseline age: 6, 9 (2) follow-up age: 14	Parent-reported -TV viewing	N-back task -Working memory	Age; sex; BMI; parental education; parental social class	TV viewing was not associated with working memory.
Wickel, (28) United States	(1) 699 (48%) (2) baseline age: 9 follow-up age: 15	Accelerometer -Sedentary time	Weinberger adjustment inventory -Inhibitory control Operation span task -Working memory Tower of London task -Planning	Ethnicity; change in PA; BMI z-score; SES	The increase in sedentary time from 9 to 15 years predicted higher inhibitory control, working memory, and planning.

*BMI, body mass index; CANTAB, Cambridge Neuropsychological Test Automated Battery; IMD, Indices of Multiple Deprivation; IQ, intelligence quotient; PA, physical activity; MVPA, moderate to vigorous physical activity; SES: socioeconomic status*.

## Discussion

The present study was aimed to critically review the evidence on the association between sedentary behavior and executive function in children and adolescents. Out of the 16 studies, seven studies (44%) found a negative association between sedentary behavior and executive function, while two studies (13%) presented positive associations. Eight studies measured sedentary time using an accelerometer, and showed varied associations of objectively measured sedentary time with executive function. Nine studies measured screen-based sedentary behavior, of which five studies (56%) found negative associations of sedentary time with executive function.

Eight of the included studies objectively measured sedentary time. The current review presented mixed results regarding the associations between objectively measured sedentary time and executive function in children and adolescents. It is impossible to conclude of the direction of the association between objectively measured sedentary time and executive function. Our findings are inconsistent with a systematic review in older adults, which indicated that shorter objectively assessed sedentary time was associated with better global cognitive function (44). Although the accelerometer-based measurements provided an objectively assessed sedentary time, they cannot distinguish the types of sedentary behavior. Children and adolescents may engage in cognitively active sedentary behavior, such as reading, and learning, benefiting cognitive development (45). A study further supports this idea. Brain connectivity was positively correlated with reading time and negatively correlated with screen-based media time (46). Therefore, when it comes to the associations between sedentary behavior and executive function in childhood, the types of sedentary behavior should be considered.

In this systematic review, nine included studies surveyed screen-based sedentary behavior, which provided some evidence on the association between type-specific sedentary behavior and executive function in children and adolescents. The majority of evidence suggests that screen-based sedentary time has either no effects or a detrimental effect on executive function in children and adolescents. Recent evidence found that the deleterious effects of sedentary behavior on cardio-metabolic health are most notable for screen-based behaviors (47). Regarding mental health, a study showed that only leisure screen-based sedentary behaviors are linked to worse perceived stress and anxiety (48). In the current study, the negative associations of sedentary behavior with executive function are mainly observed in the included studies that measured screen-based sedentary behaviors (i.e., TV viewing, computer use, video games, total screen time). These findings are in line with the systematic review in early childhood. Specifically, the systematic review concluded that screen time was either not associated with or had detrimental associations with cognitive function in young children (17).

The biological plausibility for the observed negative association of screen-based sedentary behavior with executive function is not clear. There might be several potential explanations. First, most digital screens are backlit and emit blue light wavelengths. It can suppress melatonin secretion to influence sleep quality (49, 50), which may, in turns, affect brain health (51, 52). Second, sedentary behavior may increase the risk of some aspects of mental problems, such as depression (14), which may negatively influence cognitive development (53). Third, recent neuroimaging studies have linked screen-based sedentary behavior with brain structure and integrity, which further supports a detrimental effect of screen-based sedentary behavior. A study indicated that prolonged time on TV viewing was associated with lower gray matter volume in six brain regions in children (54). Increased screen-based media use was also associated with lower microstructural integrity of brain white matter in preschool-aged children (55).

Most of the included studies were of low to moderate quality. Of the 16 studies, four studies employed longitudinal study design, and only one study was rated as strong quality. Therefore, more studies with stronger design are warranted to further ascertain the effects of sedentary behavior on cognitive function in childhood. In addition, previous studies have suggested that physical activity and exercise were positively associated with executive function in children and adolescents (18, 56). Sedentary behavior may also correlate with physical activity considering the 24-h movement continuum. However, of the 16 studies, most studies did not consider physical activity as a potential covariate. The results may have been subjected to residual confounding. Future studies should consider physical activity as covariates or investigate the combined effects of sedentary behavior and physical activity.

Although the conflicting results exist, this systematic review provided preliminary evidence which supports a negative association between screen-based sedentary behavior and executive function in children and adolescents. Therefore, from the perspective of children's physical health and cognitive development, families, schools, and policymakers should consider interventions for reducing and limiting screen-based sedentary behavior in childhood.

To the best of our knowledge, this study is the first to systematically review the associations between sedentary behavior and executive function in children and adolescents. However, this study also has its limitations. First, a meta-analysis cannot be carried out due to the heterogeneities in study design and outcome measurements of the included studies. Second, all of the included studies were observational in design, and there was no intervention study. Therefore, the causal relationship between sedentary behavior and executive function cannot be inferred. Third, the searching language was limited to English, which increases the risk of omitting important studies published in other languages.

## Conclusion

The study suggests that the associations between sedentary behavior and executive function are not conclusive in children and adolescents. However, time on screen-based sedentary behavior tends to be negatively associated with executive function.

## Data Availability Statement

The original contributions presented in the study are included in the article/Supplementary Material, further inquiries can be directed to the corresponding author/s.

## Author Contributions

SL and TH: conceptualization, writing, and original draft preparation. SL, TH, KZ, and JG: methodology and validation. SL and JG: formal analysis. SL, MS, and JG: resources. SL, JG, and MS: data curation. SL, KZ, and TH: writing—review and editing. TH: supervision and project administration. All authors have read and agreed to the published version of the manuscript.

## Conflict of Interest

The authors declare that the research was conducted in the absence of any commercial or financial relationships that could be construed as a potential conflict of interest.

## Publisher's Note

All claims expressed in this article are solely those of the authors and do not necessarily represent those of their affiliated organizations, or those of the publisher, the editors and the reviewers. Any product that may be evaluated in this article, or claim that may be made by its manufacturer, is not guaranteed or endorsed by the publisher.
