# Supplementary material for: Is Sedentary Behavior Associated With Executive Function in Children and Adolescents? A Systematic Review
Source: Front Public Health. 2022 Feb 2;10:832845. doi: 10.3389/fpubh.2022.832845 (PMC8847290; doi:10.3389/fpubh.2022.832845)
Supplement: Supplementary file 1 [file Data_Sheet_1.docx]

Supplementary Material

# Supplementary Table 1. Detailed scores of study quality assessment

| Items | 1 | 2 | 3 | 4 | 5 | 6 | 7 | 8 | 9 | 10 | 11 | 12 | 13 | 14 | Scores | Quality |
| --- | --- | --- | --- | --- | --- | --- | --- | --- | --- | --- | --- | --- | --- | --- | --- | --- |
| Aadland et al. | Y | Y | Y | Y | N | N | N | Y | Y | NA | Y | NA | NA | Y | 8/11 | Good |
| Chetty-Mhlanga et al. | Y | Y | NR | N | N | N | N | Y | Y | NA | Y | NA | NA | Y | 6/11 | Poor |
| Mora-Gonzalez et al.^2019a^ | Y | Y | NR | N | N | N | N | Y | Y | NA | Y | NA | NA | Y | 6/11 | Poor |
| Mora-Gonzalez et al.^2019b^ | Y | Y | NR | N | N | N | N | Y | Y | NA | Y | NA | NA | Y | 6/11 | Poor |
| Mora-Gonzalez et al.^2020^ | Y | Y | NR | N | N | N | N | Y | Y | NA | Y | NA | NA | Y | 6/11 | Poor |
| Fairclough et al. | Y | Y | Y | N | N | N | N | Y | Y | NA | Y | NA | NA | Y | 7/11 | Fair |
| Ribner et al. | Y | Y | NR | Y | Y | N | N | Y | Y | NA | Y | NA | NA | Y | 8/11 | Good |
| Rosenqvist et al. | Y | Y | Y | NR | N | N | N | Y | Y | NA | Y | NA | NA | Y | 7/11 | Fair |
| Syvaoja et al. | Y | Y | N | Y | N | N | N | Y | Y | NA | Y | NA | NA | Y | 7/11 | Fair |
| van der Niet et al. | Y | Y | NR | NR | N | N | N | Y | Y | NA | Y | NA | NA | Y | 6/11 | Poor |
| Verburgh et al. | Y | Y | NR | N | Y | N | N | Y | Y | NA | Y | NA | NA | Y | 7/11 | Fair |
| Xu et al. | Y | Y | NR | N | N | N | N | Y | Y | NA | Y | NA | NA | Y | 6/11 | Poor |
| Dubuc et al. | Y | Y | N | Y | N | Y | Y | Y | Y | Y | Y | NR | Y | Y | 11/14 | Good |
| López-Vicente et al. | Y | Y | NR | Y | N | Y | Y | N | Y | N | Y | NR | N | Y | 8/14 | Poor |
| O'Connor et al. | Y | Y | Y | Y | N | Y | Y | Y | Y | Y | Y | Y | N | Y | 12/14 | Strong |
| Wickel. | Y | N | NR | Y | N | Y | Y | Y | Y | Y | Y | NR | N | Y | 9/14 | Fair |

Y: yes; N: no; NA: not applied; NR: not reported; CD: cannot determine;

1) Was the research question or objective in this paper clearly stated?; 2) Was the study population clearly specified and defined?; 3) Was the participation rate of eligible persons at least 50%?; 4a) Were all the subjects selected or recruited from the same or similar populations (including the same time period)?; 4b) Were inclusion and exclusion for being in the study prespecified and applied uniformly to all participants?; 5) Was a sample size justification, power description, or variance and effect estimates provided?; 6) For the analyses in this paper, were the exposure(s) of interest measured prior to the outcome(s) being measured?; 7) Was the timeframe sufficient so that one could reasonably expect to see an association between exposure and outcome if it existed?; 8) For exposures that can vary in amount or level, did the study examine different levels of the exposure as related to the outcome?; 9) Were the exposure measures (independent variables) clearly defined, valid, reliable, and implemented consistently across all study participants?; 10) Was the exposure(s) assessed more than once over time?; 11) Were the outcome measures (dependent variables) clearly defined, valid, reliable, and implemented consistently across all study populations?; 12) Were the outcome assessors blinded to the exposure status of participants?; 13) Was loss to follow-up after baseline 20% or less?; 14) Were key potential confounding variables measured and adjusted statistically for their impact on the relationship between exposure(s) and outcome(s)?

Quality Rating: Poor (<60%), Fair (60-69%), Good (70-79%), and Strong (>80%).

# Supplementary Table 2. PRISMA 2009 checklist

| **Section/topic** | **#** | **Checklist item** | **Reported on page #** |
| --- | --- | --- | --- |
| **TITLE** | | |  |
| Title | 1 | Identify the report as a systematic review, meta-analysis, or both. | 1 |
| **ABSTRACT** | | |  |
| Structured summary | 2 | Provide a structured summary including, as applicable: background; objectives; data sources; study eligibility criteria, participants, and interventions; study appraisal and synthesis methods; results; limitations; conclusions and implications of key findings; systematic review registration number. | 1 |
| **INTRODUCTION** | | |  |
| Rationale | 3 | Describe the rationale for the review in the context of what is already known. | 1-2 |
| Objectives | 4 | Provide an explicit statement of questions being addressed with reference to participants, interventions, comparisons, outcomes, and study design (PICOS). | 1-2 |
| **METHODS** | | |  |
| Protocol and registration | 5 | Indicate if a review protocol exists, if and where it can be accessed (e.g., Web address), and, if available, provide registration information including registration number. | No |
| Eligibility criteria | 6 | Specify study characteristics (e.g., PICOS, length of follow-up) and report characteristics (e.g., years considered, language, publication status) used as criteria for eligibility, giving rationale. | 3 |
| Information sources | 7 | Describe all information sources (e.g., databases with dates of coverage, contact with study authors to identify additional studies) in the search and date last searched. | 3 |
| Search | 8 | Present full electronic search strategy for at least one database, including any limits used, such that it could be repeated. | 3 |
| Study selection | 9 | State the process for selecting studies (i.e., screening, eligibility, included in systematic review, and, if applicable, included in the meta-analysis). | 3 |
| Data collection process | 10 | Describe method of data extraction from reports (e.g., piloted forms, independently, in duplicate) and any processes for obtaining and confirming data from investigators. | 3 |
| Data items | 11 | List and define all variables for which data were sought (e.g., PICOS, funding sources) and any assumptions and simplifications made. | 3 |
| Risk of bias in individual studies | 12 | Describe methods used for assessing risk of bias of individual studies (including specification of whether this was done at the study or outcome level), and how this information is to be used in any data synthesis. | NA |
| Summary measures | 13 | State the principal summary measures (e.g., risk ratio, difference in means). | NA |
| **Section/topic** | **#** | **Checklist item** | **Reported on page #** |
| Synthesis of results | 14 | Describe the methods of handling data and combining results of studies, if done, including measures of consistency (e.g., I^2^) for each meta-analysis. | NA |
| Risk of bias across studies | 15 | Specify any assessment of risk of bias that may affect the cumulative evidence (e.g., publication bias, selective reporting within studies). | NA |
| Additional analyses | 16 | Describe methods of additional analyses (e.g., sensitivity or subgroup analyses, meta-regression), if done, indicating which were pre-specified. | NA |
| **RESULTS** | | |  |
| Study selection | 17 | Give numbers of studies screened, assessed for eligibility, and included in the review, with reasons for exclusions at each stage, ideally with a flow diagram. | 4 |
| Study characteristics | 18 | For each study, present characteristics for which data were extracted (e.g., study size, PICOS, follow-up period) and provide the citations. | Table 1 |
| Risk of bias within studies | 19 | Present data on risk of bias of each study and, if available, any outcome level assessment (see item 12). | NA |
| Results of individual studies | 20 | For all outcomes considered (benefits or harms), present, for each study: (a) simple summary data for each intervention group (b) effect estimates and confidence intervals, ideally with a forest plot. | 5, Table1 |
| Synthesis of results | 21 | Present results of each meta-analysis done, including confidence intervals and measures of consistency. | NA |
| Risk of bias across studies | 22 | Present results of any assessment of risk of bias across studies (see Item 15). | NA |
| Additional analysis | 23 | Give results of additional analyses, if done (e.g., sensitivity or subgroup analyses, meta-regression [see Item 16]). | NA |
| **DISCUSSION** | | |  |
| Summary of evidence | 24 | Summarize the main findings including the strength of evidence for each main outcome; consider their relevance to key groups (e.g., healthcare providers, users, and policy makers). | 5-7 |
| Limitations | 25 | Discuss limitations at study and outcome level (e.g., risk of bias), and at review-level (e.g., incomplete retrieval of identified research, reporting bias). | 7 |
| Conclusions | 26 | Provide a general interpretation of the results in the context of other evidence, and implications for future research. | 6-7 |
| **FUNDING** | | |  |
| Funding | 27 | Describe sources of funding for the systematic review and other support (e.g., supply of data); role of funders for the systematic review. | 7 |

NA: not applicable.

*From:*  Moher D, Liberati A, Tetzlaff J, Altman DG, The PRISMA Group (2009). Preferred Reporting Items for Systematic Reviews and Meta-Analyses: The PRISMA Statement. PLoS Med 6(7): e1000097. doi:10.1371/journal.pmed1000097

# Supplementary 3. Search strategy

Database: PubMed, Web of Science, PsycINFO, SPORTDiscus

Limitation:

1) publication date: inception to April 30, 2021; 2) language: English; 3) document type: journal/article.

Results:

Web of Science 943 results (April 30, 2021)

PubMed 178 results (April 30, 2021)

PsycINFO 9 results (April 30, 2021)

SPORTDiscus 19 results (April 30, 2021)

Keyword term:

1) sedentary behavi*, screen time, sitting time, sedentary time, TV viewing, video gam*, computer use.

2) executive function, cognitive control, working memory, inhibitory control, cognitive flexibility, planning.

3) children, adolescents.

Search strategy for each database

1# Search formula of PubMed

("sedentary behavi*"[Title/Abstract] OR "screen time"[Title/Abstract] OR "sitting time"[Title/Abstract] OR "sedentary time"[Title/Abstract] OR "TV viewing"[Title/Abstract] OR "video gam*"[Title/Abstract] OR "computer use"[Title/Abstract]) AND ("executive function"[Title/Abstract] OR "cognitive control"[Title/Abstract] OR "working memory"[Title/Abstract] OR "inhibitory control "[Title/Abstract] OR "cognitive flexibility"[Title/Abstract] OR planning[Title/Abstract]) AND (children[Title/Abstract] OR adolescents[Title/Abstract])

2# Search formula of Web of science

(TS=(“sedentary behavi*” OR “screen time” OR “sitting time” OR “sedentary time” OR “TV viewing” OR “video gam*” OR “computer use” ) And TS= ( “executive function” OR “cognitive control” OR “working memory” OR “inhibitory control” OR “cognitive flexibility” OR planning)) AND TS=(children OR adolescents)

3# Search formula of PsycINFO

Line1 [Abstract]: "sedentary behavi*" or "sedentary time" or "screen time" or "sitting time" or "TV viewing" or "video gam*" or "computer use"

Line2 [Abstract]: "executive function" or "cognitive control" or "inhibitory control" or "working memory" or "cognitive flexibility" or planning

Line3 [Abstract]: children or adolescents

4# Search formula of SPORTDiscus

Line1 [Abstract]: "sedentary behavi*" or "sedentary time" or "screen time" or "sitting time" or "TV viewing" or "video gam*" or "computer use"

Line2 [Abstract]: "executive function" or "cognitive control" or "inhibitory control" or "working memory" or "cognitive flexibility" or planning

Line3 [Abstract]: children or adolescents
